# Antioxidant N-Acetylcysteine Attenuates the Reduction of Brg1 Protein Expression in the Myocardium of Type 1 Diabetic Rats

**DOI:** 10.1155/2013/716219

**Published:** 2013-06-18

**Authors:** Jinjin Xu, Shaoqing Lei, Yanan Liu, Xia Gao, Michael G. Irwin, Zhong-yuan Xia, Ziqing Hei, Xiaoliang Gan, Tingting Wang, Zhengyuan Xia

**Affiliations:** ^1^Department of Anaesthesiology, The University of Hong Kong, HKSAR, Hong Kong; ^2^Department of Anaesthesiology, Renmin Hospital of Wuhan University, Wuhan 430060, China; ^3^Department of Anesthesiology, 3rd Affiliated Hospital of Sun Yat-sen University, Guangzhou 510630, China; ^4^Department of Anesthesiology and Critical Care, Union Hospital, Tongji Medical College, Huazhong University of Science and Technology, 1277 Jiefang Avenue, Wuhan, China; ^5^Department of Anesthesiology, Affiliated Hospital of Guangdong Medical College, Zhanjiang 524001, China

## Abstract

Brahma-related gene 1 (Brg1) is a key gene in inducing the expression of important endogenous antioxidant enzymes, including heme oxygenase-1 (HO-1) which is central to cardioprotection, while cardiac HO-1 expression is reduced in diabetes. It is unknown whether or not cardiac Brg1 expression is reduced in diabetes. We hypothesize that cardiac Brg1 expression is reduced in diabetes which can be restored by antioxidant treatment with N-acetylcysteine (NAC). Control (C) and streptozotocin-induced diabetic (D) rats were treated with NAC in drinking water or placebo for 4 weeks. Plasma and cardiac free15-F2t-isoprostane in diabetic rats were increased, accompanied with increased plasma levels of tumor necrosis factor-alpha (TNF-alpha) and interleukin 6 (IL-6), while cardiac Brg1, p-STAT3 and HO-1 protein expression levels were significantly decreased. Left ventricle weight/body weight ratio was higher, while the peak velocities of early (E) and late (A) flow ratio was lower in diabetic than in C rats. NAC normalized tissue and plasma levels of 15-F2t-isoprostane, significantly increased cardiac Brg1, HO-1 and p-STAT3 protein expression levels and reduced TNF-alpha and IL-6, resulting in improved cardiac function. In conclusion, myocardial Brg1 is reduced in diabetes and enhancement of cardiac Brg1 expression may represent a novel mechanism whereby NAC confers cardioprotection.

## 1. Introduction

Diabetes mellitus-induced cardiovascular complication is a growing life-threatening disease worldwide. Diabetic cardiomyopathy (DCM) is a diabetes-associated ventricular dysfunction, resulted from abnormal ventricular structural alteration that is independent of other etiological factors such as hypertension [1]. Studies have shown that hyperglycemia-induced oxidative stress and the subsequent inflammation play critical roles in the development and progression of diabetic cardiomyopathy [2, 3]. Hyperglycemia-induced oxidative stress mainly results from increased production of reactive oxygen species (ROS) with or without concomitantly damped antioxidant defense system [4, 5]. Our previous study found that the major endogenous antioxidant enzyme superoxide dismutase (SOD), which plays an important role in balancing ROS generation and the overall tissue antioxidant capacity, was increased in both the plasma and heart tissue of rats at a relatively early stage (4 weeks) of diabetes, but tissue and plasma levels of free 15-F_2t_-isoprostane, a specific marker of lipid peroxidation, were also increased [6]. This indicates that the upregulation of SOD is not sufficient to resist hyperglycemia-induced oxidative stress. Of note, another important enzyme, heme oxygenase-1 (HO-1), a stress-inducible cytoprotective defense enzyme, has been shown to exert cytoprotective effect against oxidative insults [7]. Also, studies showed that enhancing myocardial HO-1 expression could attenuate diabetes-induced cardiac dysfunction. However, in contrast to the compensatory increase of SOD during early diabetes mellitus, a number of studies showed that myocardial HO-1 expression was significantly decreased in the myocardium of diabetic rats [8, 9]. Thus, unveiling the underlying mechanisms governing the reduction of myocardial HO-1 in diabetes should lead to the development of novel therapies to upregulate HO-1 expression in diabetic heart.

It has been reported that, in response to oxidative stress, Brahma-related gene 1 (Brg1) is necessarily required in Nuclear factor-E2 related factor 2 (Nrf2)/ARE-mediated induction of HO-1 [10]. Brg1 is the core ATPases in the SWI/SNF complex, which plays a central role in the activation and transcription of genes in mammalian cells [11]. The deficiency of Brg1 results in the dissolution of discrete heterochromatin domains, aberrant mitotic progression, and genomic instability, which eventually induces cell death or cell apoptosis [12]. A recent study showed that Brg1 can bind to the promoters of antioxidant defense genes and protect cells from oxidative damage [13], which means that Brg1 can exert antioxidative effect. Furthermore, increasing evidence shows that Brg1 can regulate gene expression during cardiac growth, differentiation, and hypertrophy [14–16]. Brg1 null mice embryos die when cardiomyocytes expansion and maturation begin, while in adult cardiomyocytes, Brg1 is activated by cardiac stresses and assembles a chromatin complex to activate downstream signal transduction, including HO-1 and signal transducer and activator of transcription 3 (STAT3) [10, 16–18], which are protective against ROS-induced cardiomyopathy. Studies by us and others found that myocardial HO-1 expression is reduced in diabetic rats [8, 9] which is accompanied with reduced phosphorylation of STAT3 (p-STAT3, the activated status of STAT3) [19]. We postulated that reductions in myocardial HO-1 and STAT3 in diabetes may be a consequence of reduction in cardiac Brg1 expression subsequent to hyperglycemia-induced oxidative stress.

Accumulated evidence proves that therapies that can reduce oxidative stress are effective to attenuate the development of diabetic cardiomyopathy [20, 21]. Our previous study also found that treatment with the antioxidant N-acetylcysteine (NAC) could attenuate the increase in inflammation factors tumor necrosis factor-alpha (TNF-alpha) and interleukin 6 (IL-6) [6] and ameliorate myocardial dysfunction [2] in diabetic rats. Moderate levels of TNF-alpha or IL-6 have been shown to initiate the activation of STAT3, a key protein in cardioprotective signaling pathway, whose activation is Brg1 dependent [22, 23]. Therefore, the current study was designed to test the hypothesis that myocardial Brg1 is reduced in diabetes and antioxidant NAC may enhance cardiac Brg1 expression and concomitantly increase cardiac STAT3 activation and confer cardioprotection in diabetes.

## 2. Materials and Methods

### 2.1. Animals and Introduction of Diabetes

Sprague-Dawley male rats (220 ± 20 g, 8 weeks of age) were obtained from the Laboratory Animal Service Center (University of Hong Kong). All rats were allowed to adapt in their houses and have free access to standard chow and water according to the principles of Animal Care of the University of Hong Kong. The experiment procedures were approved by the Committee on the Use of Live Animals in Teaching and Research (CULATR). Diabetes was induced by a single tail vein injection of streptozotocin (STZ) at the dose of 65 mg/kg body weight (Sigma-Aldrich, St. Louis, MO), freshly dissolved in 0.1 M citrate buffer (PH 4.5) under anesthesia with sodium pentobarbital (65 mg/kg body weight), while control rats were given equal volume of citrate buffer alone. After three days of injection, blood glucose was measured using a Glucose Analyzer (Bayer Healthcare, Bayer AG, Germany), and rats with blood glucose over 16.7 mM were considered diabetic.

### 2.2. Experimental Protocol

Rats were randomly divided into three groups (*n* = 6 per group): control (C); diabetes (D); diabetes treated with NAC (1.5 g/kg/day) (D + NAC). NAC was administered to the D + NAC group dissolved in drinking water for 4 weeks after induction of diabetes starting one week after the onset of diabetes. Upon completion of treatment, the rats were anticoagulated with heparin (1000 IU/kg) and then anaesthetized with pentobarbital sodium (65 mg/kg body weight). Blood samples were obtained from the inferior vena cava, and plasma was separated and stored at −80°C for further analysis. Rats were sacrificed after completion of echocardiographic assessment of cardiac function, and hearts were harvested and rinsed with ice-cold phosphate buffer saline, dried, and weighted.

### 2.3. Echocardiography

At the end of 4-week treatment, the rats were examined by echocardiography using a High Resolution Imaging System (Vevo 770, VisualSonics Inc., Canada) equipped with a 17.5-MHz liner array transducer. The following validated parameters were automatically calculated by the ultrasound machine: LV end-diastolic volumes (LVVd), LV end-systolic volume (LVVs), fractional shortening (FS), ejection fraction (EF), and stroke volume (SV). M-mode images were recorded to detect heart rate (HR), LV internal diameter in systole (LVIDs) and diastole (LVIDd), interventricular septal thickness in systole (IVSs) and in diastole (IVSd), and LV posterior wall thickness in systole (LVPWs) and diastole (LVPWd). LV mass was assessed by calculating the formula: LV mass = 1.053 [(LVIDd + LVPWd + IVSd)^3^ − LVIDd^3^] × 0.8. The peak velocities of early (E) and late (A) flows were obtained from the apical four-chamber view. The E/A ratio and the isovolumetric relaxation time (IVRT) were used as indices of LV diastolic function.

All recordings were performed in rats that underwent inhalation of 3% isopentane in air throughout the whole process, and echocardiography was conducted by investigators who were blinded to the experimental group as we reported [24].

### 2.4. Measurement of Free 15-F_2t_-Isoprostane, TNF-Alpha, and IL-6

Free 15-F_2t_-isoprostane (15-F_2t_-IsoP), a specific marker of oxidative stress, was measured by using an enzyme-linked immunoassay kit (Cayman chemical, Ann Arbor, MI) as described [20]. Plasma samples and homogenized heart tissue (in PBS) were purified using Affinity Sorbent and Affinity Column (Cayman chemical, Ann Arbor, MI), then processed for analysis, according to the protocol provided by the manufacturer. The values of plasma or cardiac free 15-F_2t_-IsoP were expressed as pg/mL in plasma or pg/mg protein in cardiac homogenates, respectively.

Plasma levels of TNF-alpha and IL-6 were determined by using the commercially available rat ELISA kit (Bender Med, Vienna, Austria).

### 2.5. Western Blot Assay for HO-1, STAT3, and p-STAT3

Frozen heart tissue was homogenized using lysis buffer (20 mmol/L Tris-HCl PH = 7.5, 50 mmol/L 2-mercaptoethanol, 5 mmol/L EGTA, 2 mmol/L EDTA, 1% NP40, 0.1% sodium dodecyl sulfonate (SDS), 0.5% deoxycholic acid, 10 mmol/L NaF, 1 mmol/L PMSF, 25 mg/mL leupeptin, and 2 mg/mL aprotinin) for 30 min and then sonicated and centrifuged at 12000 g for 20 min at 4°C. Protein concentrations were determined using the Bradford assay (Bio-Rad, USA). Samples containing equal amounts were separated on a 10% SDS-polyacrylamide gel, and then proteins were transferred to PVDF membrane overnight at 4°C. Membranes were blocked with 5% nonfat milk in Tris-Buffered Saline (TBS)-Tween for 1 hour and were incubated with anti-Brg1 (Abcam, USA) or anti-STAT3, anti-phospho-STAT3 (T705), anti-HO-1 antibodies (Cell Signaling Technology, Beverly, MA, USA), and GAPDH (Cell Signaling Technology, Beverly, MA, USA) at 1 : 1000 dilution for overnight at 4°C. After washing with phosphate buffered saline-tween (PBST) three times for 30 min, membranes were then incubated with horseradish-peroxidase- (HRP-) conjugated anti-rabbit IgG at 1 : 2000 dilution for 1 hour. Protein bands were developed with enzymatic chemiluminescence, and images were measured by a densitometer with analysis software.

### 2.6. Statistical Analysis

Data are presented as means ± standard error of the mean (S.E.M.). Data were analysed by the ANOVA within the same group and between groups. Multiple comparisons of group means were analyzed by Tukey's test. The analysis was performed using statistical software package (GraphPad Prism, San Diego, CA, USA). Significant difference was defined as *P* ≤ 0.05.

## 3. Results

### 3.1. General Characteristics and Effects of NAC Treatment

Administration of STZ resulted in increased plasma glucose and food and fluid intake and reduced body weight gain as compared with age-matched control rats (all *P* < 0.05, Table 1). Treatment with NAC for 4 weeks significantly reduced food consumption and water intake in diabetic rats (*P* < 0.05, D + NAC versus D) but did not have significant effect on glucose levels and body weight gain.

### 3.2. Oxidative Stress Marker Free 15-F_2t_-IsoP Levels

Compared with the control group, the levels of free 15-F_2t_-IsoP were significantly increased in both the plasma and cardiac tissues of diabetic rats (*P* < 0.01 or *P* < 0.05 versus C, Table 2). NAC treatment reduced plasma and cardiac tissue 15-F_2t_-IsoP to a level comparable to that in the control (*P* < 0.05 versus D, *P* > 0.05 D + NAC versus C, Table 2).

### 3.3. Effect of NAC on Left Ventricular Dimension and Function

As shown in Table 3, LVM was much lower in diabetic rats (*P* < 0.05 versus C), despite the fact that there were no significant differences in IVSd, IVSs, LVIDs, LVPWd, and LVPWs between the control and the diabetic rats. However, LVM to body weight ratio, an indicator of myocardial hypertrophy, was remarkably increased in diabetic rats (*P* < 0.05 versus C). NAC reduced LVM to body weight ratio to a level comparable to that in the control (*P* < 0.05, D + NAC versus D; *P* > 0.05 D + NAC versus C, Table 3). The HRs of diabetic rats were significantly decreased as compared to those of the controls (*P* < 0.05 versus C Table 3). NAC had no effect on HR. LVVd and the E/A ratio in diabetic rats were significantly decreased, while IVRT increased (all *P* < 0.01 versus C Table 3). This is indicative of compromised LV relaxation, which may contribute to the significantly reduced SV (*P* < 0.05 D versus C, Table 3). NAC treatment did not have significant effects on LVVd and IVRT nor did it improve SV (*P* > 0.05 D + NAC versus D, Table 3) but remarkably increased E/A ratio which primarily resulted from a reduction in MVA (*P* < 0.05 D + NAC versus D, Table 3). There was no difference in values of FS and EF between the control and diabetic rats.

### 3.4. Plasma Il-6 and TNF-*α*

**Figure 1:**
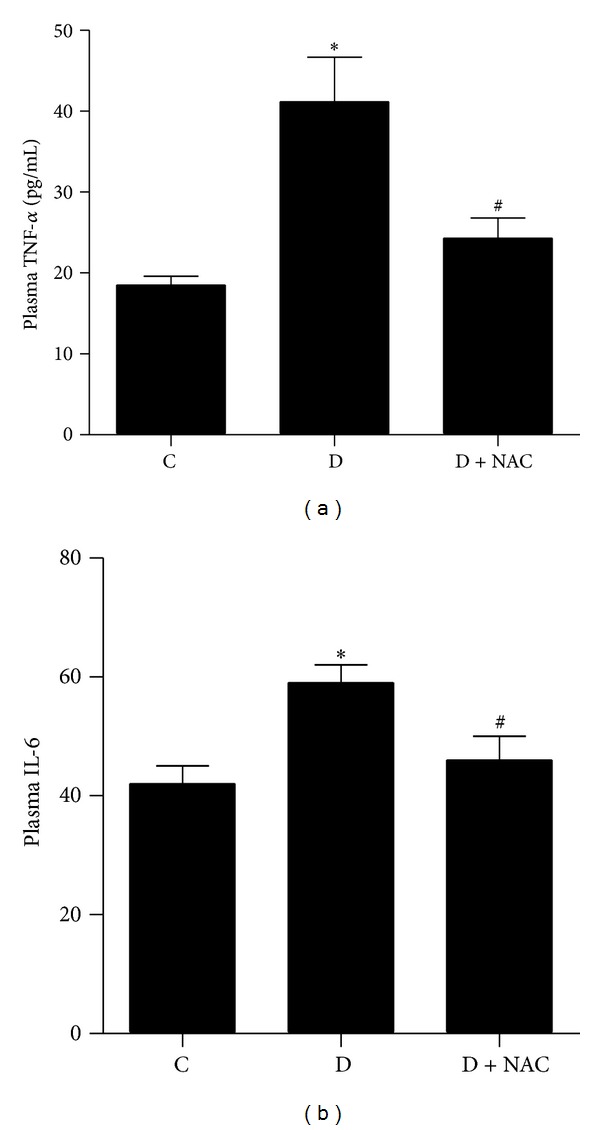
Effects of N-acetylcysteine (NAC) treatment on the level of TNF-alpha (a) and IL-6 (b) in plasma. Control (C) or STZ-induced diabetic rats without treatment (D) or with NAC treatment (1.5 g/kg/day, D + NAC) for 4 weeks. All values are expressed as mean ± S.E.M. *n* = 6 per group. *P < 0.05 versus C; ^#^ *P* < 0.05 versus D.

As shown in Figures 1(a) and 1(b), the plasma levels of TNF-*α* and IL-6 in diabetic rats were significantly increased as compared with the control group (*P* < 0.05). NAC treatment reduced plasma TNF-*α* and IL-6 to a level comparable to that in the control (*P* < 0.05 versus D; *P* > 0.05 versus C) although they were slightly higher than that in the control rats.

### 3.5. Effect of NAC on Protein Expression of Brg1

**Figure 2:**
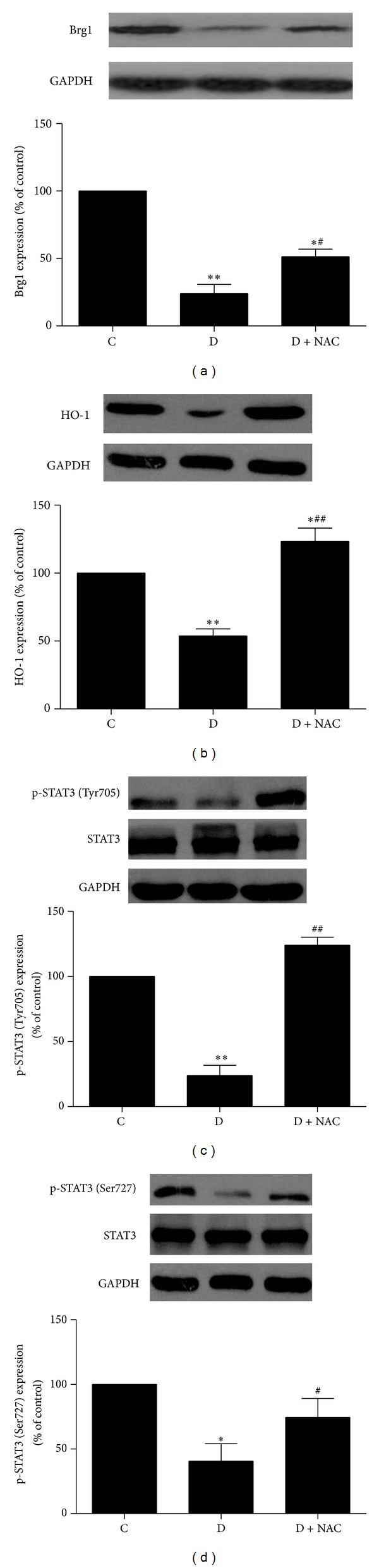
Effects of N-acetylcysteine (NAC) treatment on the protein expression of Brg1 (a), HO-1 (b), and p-STAT3 ((c), (d)) in myocardium. Control (C) or STZ-induced diabetic rats without treatment (D) or with NAC treatment (1.5 g/kg/day, D + NAC) for 4 weeks. All values are expressed as mean ± S.E.M. *n* = 6 per group. *P < 0.05, **P < 0.01 versus C; ^#^ *P* < 0.05, ^##^ *P* < 0.01 versus D.

To investigate whether the cardiac protein expression of Brg1 is altered in diabetic rats at an early stage of the disease and whether or not it can be affected by antioxidants, we explored the effects of NAC on cardiac levels of Brg1 in STZ-induced diabetic rats 4 weeks after the establishment of diabetes. As shown in Figure 2(a), the protein expression of Brg1 was significantly decreased in diabetic rats as compared to that of control group (*P* < 0.01). NAC treatment partially but significantly restored the protein expression of Brg1.

### 3.6. Effect of NAC on Protein Expression of STAT3 and HO-1

Recent study demonstrated that Brg1 is required to establish chromatin accessibility at STAT3 binding targets [18], which is essential to enable these sites to respond to downstream signaling. Therefore, in addition to exploring the changes of myocardial Brg1 protein in diabetes, we also investigated the myocardial protein levels and phosphorylation/activation status of STAT3 in diabetic heart. As shown in Figures 2(c) and 2(d), the protein expression of p-STAT3 (Tyr705 and Ser727) but not total STAT3 was significantly reduced in the myocardium of diabetic rats, accompanied with concomitant reduction of cardiac protein expression of HO-1 (all *P* < 0.05 versus C, Figure 2(b)), an important signaling protein downstream of Brg1. NAC completely restored myocardial p-STAT3 at site Tyr705 and HO-1 protein expression and partially but significantly enhanced p-STAT3 at site Ser-727 in diabetic rats (*P* < 0.01 versus D; *P* < 0.05, *P* < 0.01 versus C).

## 4. Discussion

Consistent with our previous studies, we have shown in the current study that oxidative stress increased in the early stage (at 4 weeks) rats with STZ-induced type 1 diabetes as indicated by a significant increase in both plasma and heart tissue levels of free 15-F_2t_-isoprostane, a specific index of oxidative stress [25]. Enhanced levels of oxidative stress were accompanied by increased TNF-alpha and IL-6. In the current study, we further discovered that diabetic rat hearts exhibited decreased expression of Brg1, which was coincident with decreased cardiac expressions of p-STAT3 and HO-1 and compromised cardiac diastolic function as assessed by echocardiography. Effective antioxidant treatment with NAC evidenced as complete prevention of hyperglycemia-induced increases in plasma and heart tissue free 15-F_2t_-isoprostane significantly attenuated the reduction of myocardial Brg1 protein expression, subsequently significantly enhanced myocardial p-STAT3 and HO-1, and improved cardiac relaxation in diabetic rats. To our knowledge, this is the first study to explore the changes of cardiac Brg1 in diabetic rats and the effectiveness of antioxidant treatment on Brg-1 cardiac expression in diabetes. 

Oxidative stress occurs in diabetes as a consequence of hyperglycemia-induced abnormalities, including glucose autooxidation, the formation of advanced glycation end products, and impairment of antioxidant defense system [4]. We previously reported that the heart tissue SOD activity was compensatorily increased, but both the plasma and heart tissue levels of free 15-F_2t_-isoprostanes were still increased in diabetic rats at the early stage of 4-week diabetes [6], which indicates that, during early stage of diabetes, compensatory increase in myocardial SOD was not sufficient to combat hyperglycemia-induced oxidative stress. While in our current study conducted in the same model of early stage of 4-week diabetes [6], we showed that the protein expression of HO-1, another important antioxidative enzyme, was decreased significantly in the myocardium of diabetic rats, which suggests that a decrease in HO-1 expression may be a major contributor to hyperglycemia-induced oxidative stress in early diabetes. Many studies confirmed that HO-1 plays a central role in cardiovascular protection [26]. It has been shown that, in response to oxidative stress, HO-1 expression can be induced through the Nrf2/ARE signaling pathway, while, effective Nrf2/ARE signaling needs the participation of Brg1 [10]. However, as we showed in the current study, cardiac Brg1 expression in rats at the 4th week after STZ-induced type 1 diabetes was significantly reduced, which might be the major reason why cardiac protein expression of HO-1 was significantly decreased. Antioxidant NAC normalized cardiac free 15-F_2t_-isoprostanes in diabetic rats and enhanced myocardium Brg1 expression, leading to full restoration of cardiac HO-1 expression. This finding suggests that enhancing Brg1 may represent a novel mechanism whereby NAC confers its antioxidant protection at least in early diabetes.

Consistent with our recent study findings [24], we showed in the current study that ventricular dysfunction occurs during early stage of diabetes manifested as abnormal relaxation function that was coincident with significant reduction in myocardial Brg1 protein expression. This finding is similar in nature to a previous study which showed that mice with cardiac-specific deletion of Brg1 developed impaired cardiac relaxation evidenced as reduction in E/A ratio as determined by ultrasound [15]. The findings by us and others [15] point out the importance of Brg1 in the maintenance of normal cardiac function. In our study, NAC treatment mediated improvement in cardiac diastolic function manifested as significant elevation of E/A ratio in diabetes which may be contributable to enhancement of cardiac Brg1 protein expression.

The risk of progression to heart failure after myocardial ischemia and reperfusion was significantly higher in diabetes compared with nondiabetes [27]. Myocardial STAT3 is an important transcription factor in the SAFE pathway (i.e., JAK2/STAT3 signaling cascade), especially during myocardium ischemia reperfusion injury [28], but cardiac p-STAT3 is reduced in diabetes [19]. Further, STAT3-deficient mice spontaneously develop a form of dilated cardiomyopathy similar to that which occurred in diabetic mice [29], indicating that reduced STAT3 activation may lead to myocardial remodeling. Brg1 is necessarily required to establish chromatin restructure for the activation of STAT3, especially STAT3 phosphorylation at site Try705 in various cells such as cancer cell and macrophagocyte [30], and for STAT3 signaling transduction. In the current study, we also found that STAT3 phosphorylation at both Ser727 and Try705 was dramatically reduced in the hearts of diabetic rats, which was concomitant with decreased Brg1 expression. NAC treatment increased the expression of Brg1 and consequently enhanced p-STAT3 at both Try705 and Ser727 in the myocardium of diabetic rats. Based on the fact that Brg1 is a needed component for STAT3 activation [30], our findings suggest that Brg1 may play a key role in the transcriptional induction of cardiac STAT3, especially at Try705 in diabetic rats. NAC may have increased STAT3 phosphorylation in diabetes through enhancing Brg1 expression, although further study is needed to confirm this hypothesis.

Inflammation has been considered as an important process in the progression of diabetes [31, 32]. Elevation of TNF-alpha and IL-6 was detected in diabetes, which was related to the progression of diabetic complications [33]. In the current study, we showed that plasma levels of TNF-alpha and IL-6 were remarkably elevated in diabetic rats at 4 weeks after the establishment of diabetes, and NAC treatment decreased them, indicating that NAC can inhibit the inflammation reaction in diabetes. Studies found that moderate levels of TNF-alpha or IL-6 can increase the phosphorylation of STAT3 at Try705, and this is Brg1 dependent [22, 23]. In our study, we found that the levels of TNF-alpha and IL-6 in NAC-treated diabetic rats were significantly lower than that in the untreated diabetes, but still slightly higher than that in nondiabetic rats. These remaining slight elevations in TNF-alpha and IL-6 after NAC treatment should have contributed to the enhancement of cardiac p-STAT3 as seems to be in the NAC treated group, which added to the effect of Brg1 in enhancing cardiac Brg1-mediated STAT3 activation as previously mentioned. This explains why NAC treatment did not completely restore cardiac Brg1 protein expression in diabetic rats but completely restored cardiac p-STAT3 at site Try705.

**Figure 3:**
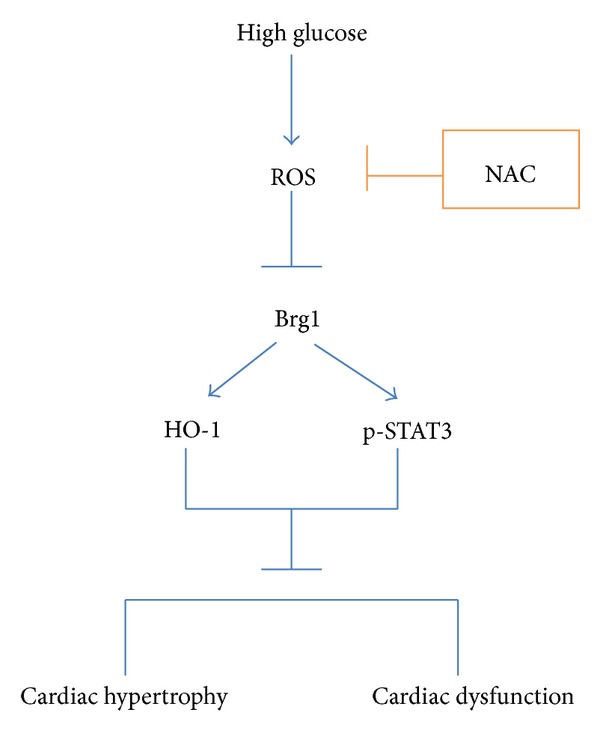
Schematic diagram proposing that Enhancement of cardiac Brg1 expression represents a novel mechanism whereby antioxidant N-acetylcysteine (NAC) enhanced cardiac HO-1 and p-STAT3 expression, and attenuated cardiac diastolic dysfunction in diabetes.

In summary, we first report that the expression of Brg1 was decreased significantly in the myocardium of diabetic rats, which may be responsible at least in part for the reduced expressions of HO-1 and p-STAT3 and impairment of cardiac diastolic function as summarized in the schematic diagram (Figure 3). Enhancement of cardiac Brg1 expression may thus represent a novel mechanism whereby NAC enhanced cardiac HO-1 and p-STAT3 expressions and attenuated cardiac diastolic dysfunction in diabetes. 

## Figures and Tables

**Table 1:** General Characteristics of Rats at the End of the Study.

	C	D	D + NAC
Water intake (mL/kg/day)	121.1 ± 8.3	840.3 ± 10.7*	421.1 ± 7.1*
Food intake (g/kg/day)	66.0 ± 1.3	195.1 ± 3.8*	145.5 ± 3.5*
Body weight (g)	486.3 ± 12.7	310.9 ± 17.2*	304.5 ± 12.5*
Plasma glucose (mM)	6.2 ± 0.8	27.7 ± 1.7*	26.1 ± 1.5*

All values are expressed as Mean ± S.E.M. *n* = 6 per group. Control (C) or STZ-induced diabetic rats with untreated (D) or treated with NAC (1.5 g/kg/day) for 4 weeks.  *P < 0.05 versus C;  ^#^ *P* < 0.05 versus D.

**Table 2:** Effects of NAC treatment on the level of free 15-F_2t_-isoprostane in plasma and heart tissue.

	C	D	D + NAC
Plasma (pg/mL)	125.1 ± 18.9	245.0 ± 19.1**	150.7 ± 21.4^#^
Heart tissue (pg/mg protein)	101.3 ± 17.3	208.5 ± 20.6*	167.2 ± 18.5^#^

All values are expressed as Mean ± S.E.M. *n* = 6 per group. Control (C) or STZ-induced diabetic rats with untreated (D) or treated with NAC (1.5 g/kg/day) for 4 weeks.  *P < 0.05 versus C;  ^#^ *P* < 0.05 versus D.

**Table 3:** M-mode Echocardiographic and transmitral Doppler flow velocity indices of LV dimensions and functions.

	C	D	NAC
LVIDd (mm)	8.30 ± 0.17	7.90 ± 0.15	7.75 ± 0.15*
LVIDs (mm)	4.80 ± 0.14	4.55 ± 0.13	4.65 ± 0.25
IVSs (mm)	2.25 ± 0.11	2.24 ± 0.08	2.08 ± 0.06
IVSd (mm)	1.72 ± 0.06	1.78 ± 0.05	1.60 ± 0.05
LVPWs (mm)	3.06 ± 0.07	2.92 ± 0.05	2.57 ± 0.05*
LVPWd (mm)	1.92 ± 0.04	1.87 ± 0.05	1.62 ± 0.03^#^
LVM (mg)	964 ± 27.5	812 ± 14.3*	707 ± 18.7*
LVM/body	1.90 ± 0.04	2.44 ± 0.07*	2.17 ± 0.04^#^
weight (mg/g)			
HR (bpm)	323 ± 7.8	285 ± 10.8*	286 ± 9.2*
LVVd (*μ*L)	372.7 ± 23.4	318.5 ± 24.2*	319.7 ± 18.4
LVVs (*μ*L)	107.8 ± 10.6	93.7 ± 9.8	101.5 ± 13.2
IVRT (ms)	21.6 ± 1.6	32.5 ± 1.8*	28.5 ± 1.4*
MV *E* (cm/s)	133.2 ± 58.3	1217 ± 30.2	1181.7 ± 62.2
MV *A* (cm/s)	909.5 ± 36.2	1028.9 ± 36.1	838.6 ± 63.8^#^
E/A	1.49 ± 0.09	1.19 ± 0.03*	1.45 ± 0.07^#^
SV (*μ*L)	277.8 ± 17.8	226.7 ± 17.4*	218.3 ± 12.2*
FS (%)	42.5 ± 0.9	42.1 ± 0.8	40.7 ± 1.0
EF (%)	71.6 ± 1.7	72.2 ± 1.7	68.8 ± 3.0

All values are expressed as Mean ± S.E.M. *n* = 6 per group. M-mode Echocardiographic and transmitral Doppler flow velocity indices of LV dimensions and functions in Control (C), Diabeties (D), Ruboxistaurin (RBX), N-acetylcysteine (NAC) rats.  *P < 0.05 or 0.01 versus C;  ^#^ *P* < 0.05 or 0.01 versus D. LVIDd: LV internal diastolic diameter; LVIDs: LV internal systolic diameter; IVSs: systolic interventricularseptal thickness; IVSd: diastolic interventricularseptal thickness; LVPWs: LV systolic posterior wall thickness; LVPWd: LV diastolic posterior wall thickness; LVM: LV mass; HR: heart rate; LVVd: LV end-diastolic volume; LVVs: LV end-systolic volume; IVRT: isovolumetric relaxation time; SV: stroke volume; FS: fractional shortening; EF: ejection fraction.
